# Patterns, Efficacy, and Cognitive Effects of Medical Cannabis Use in Chronic Musculoskeletal Pain Patients

**DOI:** 10.7759/cureus.84102

**Published:** 2025-05-14

**Authors:** Mohammad Khak, Sina Ramtin, Juliet Chung, Asif M Ilyas, Ari Greis

**Affiliations:** 1 Department of Orthopaedic Surgery, Rothman Orthopaedic Institute, Philadelphia, USA; 2 Department of Orthopaedic Surgery, Rothman Orthopaedic Institute Foundation for Opioid Research & Education, Philadelphia, USA; 3 Department of Orthopaedic Surgery, Penn State College of Medicine, Hershey, USA; 4 Department of Orthopaedics, Rothman Orthopaedic Institute, Thomas Jefferson University, Philadelphia, USA; 5 Department of Medical Cannabis, Rothman Orthopaedic Institute, Thomas Jefferson University, Philadelphia, USA

**Keywords:** cannabis, chronic pain management, medical cannabis, musculoskeletal pain, pain management, patient-reported outcome measures

## Abstract

Background: Medical cannabis (MC) is being used with greater frequency in the management of chronic pain. While its efficacy in pain relief is promising, questions about patterns of use and efficacy warrant further investigation. This study aimed to evaluate long-term MC use patterns, perceived efficacy, and its impact on cognition among patients with chronic musculoskeletal noncancer pain.

Methods: This prospective study included patients who were certified for MC between October 2022 and December 2024. Patients who were certified for MC under Pennsylvania state guidelines for a minimum of one year were tracked, yielding 129 patients for analysis. The patients completed an Inventory of Medical Cannabis Use (IMCU) questionnaire assessing usage patterns, dosage knowledge, efficacy, cognitive effects, and tolerance changes. The responses were collected in a password-protected database.

Results: A total of 77.5% of patients reported using MC daily or near daily. Topical formulations were most frequently used (63.6%). Approximately half of the respondents were uncertain of their exact tetrahydrocannabinol/cannabidiol (THC/CBD) dosage, with a median oral dose of 10 mg recorded among those who provided estimates. High levels of perceived efficacy were reported, with over 93% of respondents agreeing or strongly agreeing that MC improved their primary symptoms. Cognitive and motor effects were minimal for most users, with 72.1% reporting no impact. Furthermore, 79.8% of respondents indicated stable usage patterns over the prior three months, and very few reported a need or external suggestion to reduce MC intake.

Conclusions: Long-term MC use is a stable and well-tolerated option for managing chronic musculoskeletal pain, with high patient-reported efficacy and minimal cognitive impact. These findings support its role in pain management while highlighting the need for further research on optimal dosing and long-term safety.

## Introduction

The therapeutic application of medical cannabis (MC) has raised global attention, yet a comprehensive understanding of its efficacy and side effects remains a pivotal aspect of its integration into clinical practice. Cannabis and its derivatives show promise in treating chronic pain by acting on CB1 receptors, believed to modulate pain signals in the brain, and CB2 receptors in the dorsal root ganglion that influence pain integration in nerve pathways [1]. Cannabinoid receptors CB1 and CB2 are part of the endocannabinoid system; CB1 receptors are primarily located in the central nervous system and modulate pain perception, mood, and appetite, while CB2 receptors are found mainly in immune cells and are involved in regulating inflammation and immune responses. Patients using MC have reported significant relief from chronic pain, surpassing 50% compared to a placebo [2]. While MC can offer pain relief, its effects on cognition are notable, particularly on executive function, memory, and attention. Short-term memory impairment is common and exacerbates with chronic use, accompanied by reduced effort, slower processing, and impacted attention. Long-term and early-age use are commonly associated with neurocognitive deficits, supported by neuroimaging studies showing reduced hippocampal volume and density [3].

Despite the growing acceptance of MC as a therapeutic option for chronic musculoskeletal pain, significant gaps remain in understanding its long-term efficacy, patterns of use, and potential cognitive effects. Furthermore, the lack of standardized dosing guidelines and the variability in individual responses highlight the need for healthcare provider recommendations and a more tailored approach to MC therapy. This study aims to address these gaps by analyzing patterns of MC use, patient-reported outcomes, and the cognitive and functional effects associated with its consumption.

## Materials and methods

Institutional review board approval was obtained before the initiation of data collection. Physicians participating in MC certification completed a mandatory four-hour continuing medical education course and sought accreditation from the Pennsylvania Department of Health. To qualify for MC certification, patients were carefully screened to confirm Pennsylvania residency and diagnosis with one of the 23 state-approved medical conditions [4].

Patients diagnosed with chronic musculoskeletal noncancer pain who were certified for medical cannabis at the Rothman Orthopaedic Institute's Medical Cannabis Department between October 2022 and December 2024 were prospectively enrolled. Following their initial certification, they attended annual follow-up visits, during which they completed the Inventory of Medical Cannabis Use (IMCU) questionnaire (Appendix). This questionnaire assessed cannabis type, delivery method, dosage, side effects, and tolerance concerns. Data from the IMCU responses were securely stored in a password-protected database. A total of 129 long-term MC users completed the IMCU, providing insights into usage patterns, product preferences, dosage awareness, efficacy, cognitive effects, and tolerance changes. The median age of patients was 65 years (range: 20-91), with women comprising 84 out of 129 participants (65.1%). Detailed occupational data were not consistently reported across studies and, therefore, could not be reliably analyzed.

The patient certification process involved confirmation of one of the state-approved medical conditions and a review of their mental health and pain management history. Individuals eligible for MC underwent a chart review to identify any significant history of substance abuse or severe mental health disorders. Those patients were subsequently excluded. Additionally, the eligible patients’ prescription-controlled substance use history was reviewed using Pennsylvania’s Prescription Drug Monitoring Program. If a patient was currently prescribed opioids, their prescribing physician was involved to discuss the introduction of MC in their treatment plan. During the certification visit, patients received detailed information on the chemical constituents of MC, various delivery methods, optimal dosing parameters, and recommended formulations by the certifying physicians. Once certified, patients could obtain an MC identification card from the Pennsylvania Department of Health, granting access to purchases at authorized cannabis dispensaries within the state.

## Results

**Figure 1:**
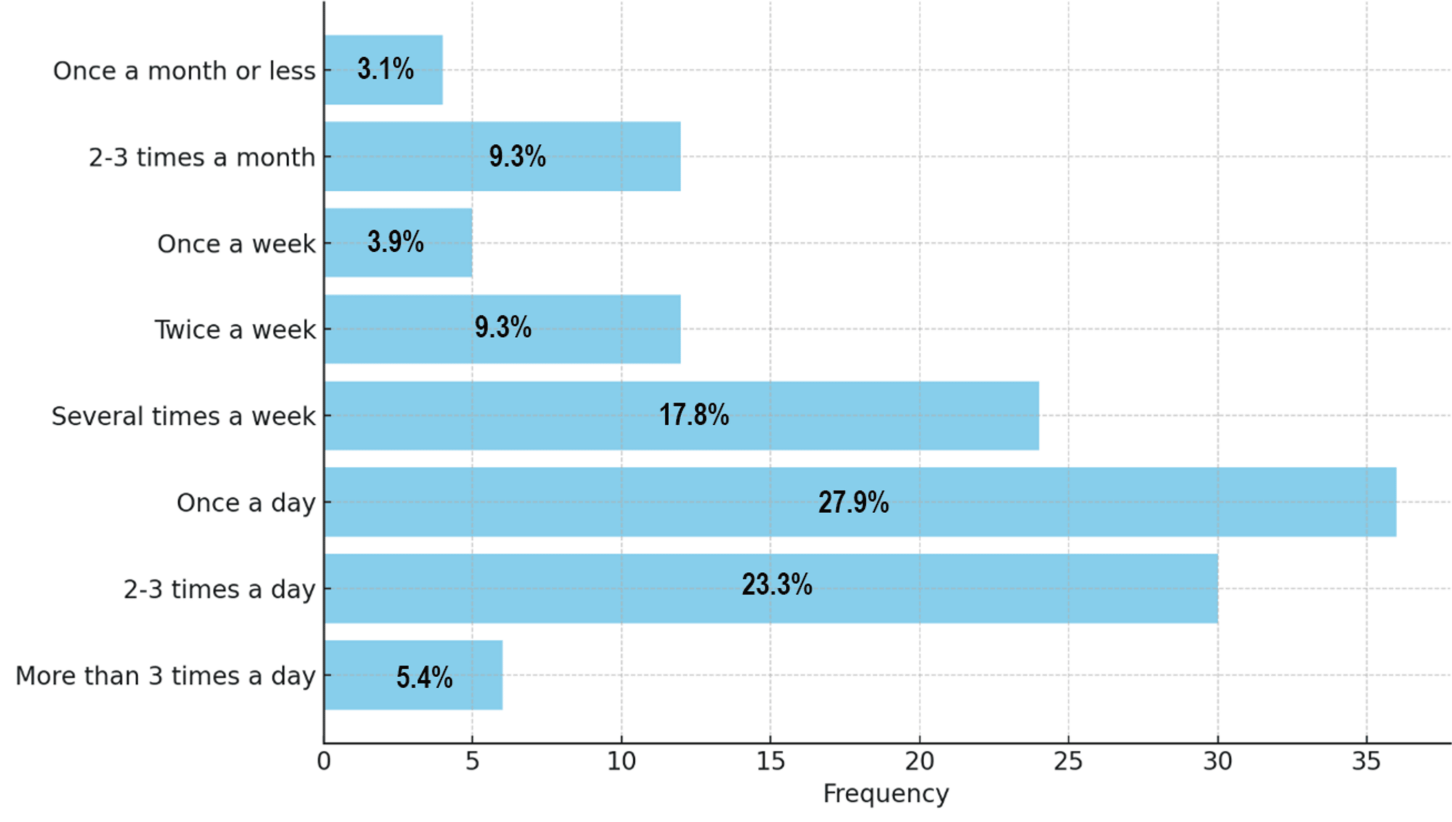
Frequency of cannabis use. Data are presented as the number and percentage of respondents (N, %).

The IMCU questionnaire, administered to 129 respondents, revealed that the majority (100/129; 77.5%) reported using cannabis for more than two years, while 29 respondents (22.5%) had used it for more than one year but less than two years. Most respondents reported using cannabis once a day (36/129; 27.9%) or two to three times a day (30/129; 23.2%). A smaller number (4/129; 3.1%) reported using it once a month or less (Figure 1).

**Figure 2:**
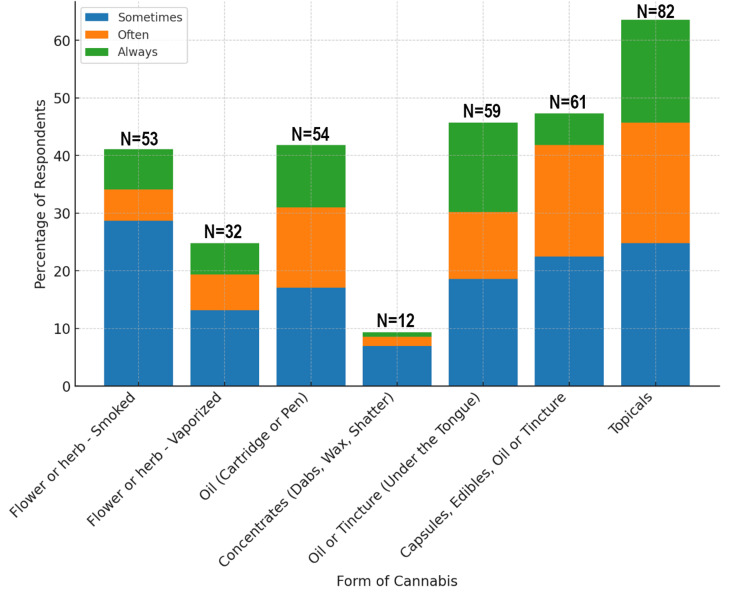
Forms of cannabis used and the frequency of use. Data are shown as the number and percentage of respondents (N, %). Categories indicate frequency of use: sometimes, often, and always.

Topicals were the most consistently used form of cannabis, reported by 82 respondents (63.5%). Capsules, edibles, or tinctures were also frequently used, with 59 respondents (45.7%) selecting oil or tincture under the tongue, and 61 (47.2%) consistently using capsules, edibles, oil, or tinctures. In contrast, more intense forms, such as concentrates (e.g., dabs, wax, and shatter), were used by only 12 respondents (9.3%) (Figure 2).

**Figure 3:**
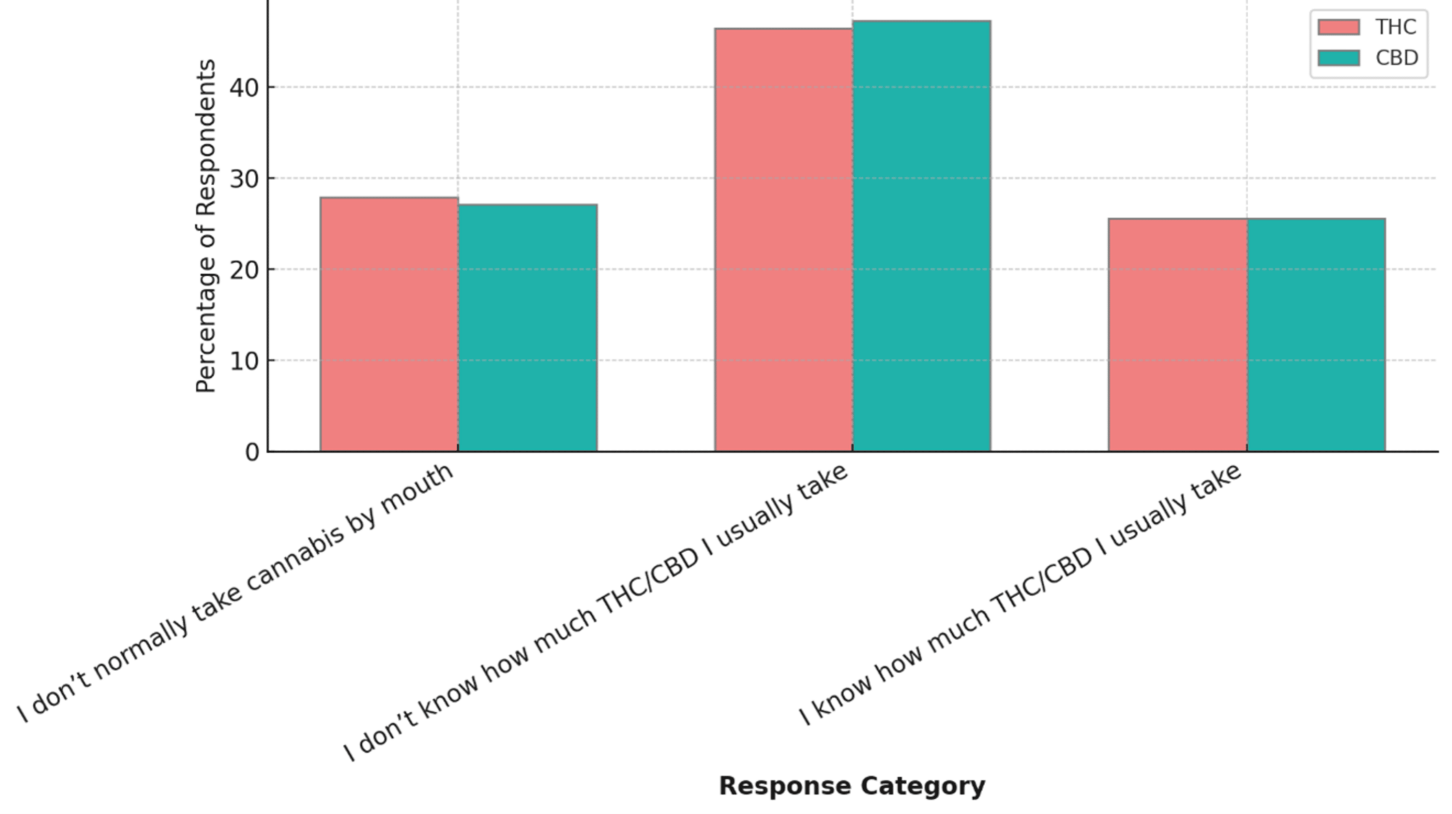
Amount of THC and CBD taken by mouth (edibles, tinctures, capsules, etc.). Data are represented as the percentage of respondents (%). CBD: cannabidiol; THC: tetrahydrocannabinol.

Respondents were asked about the amount of cannabidiol (CBD) and tetrahydrocannabinol (THC), two of the most well-known active compounds found in the cannabis plant, they consume when using cannabis by mouth (via edibles, tinctures, or capsules). For THC, 60/129 (46.5%) did not know their usual dose, and 36/129 (27.9%) reported not taking cannabis by mouth. Only 33/129 (25.5%) knew their typical oral THC dosage. For CBD, 61/129 (47.2%) were unaware of the amount they consumed, 35/129 (27.1%) did not take it by mouth, and 33/129 (25.5%) knew the dosage (Figure 3). The median THC/CBD dose taken by mouth was 10 mg, while most respondents take smaller doses, with a few outliers skewing the mean higher than the median.

**Figure 4:**
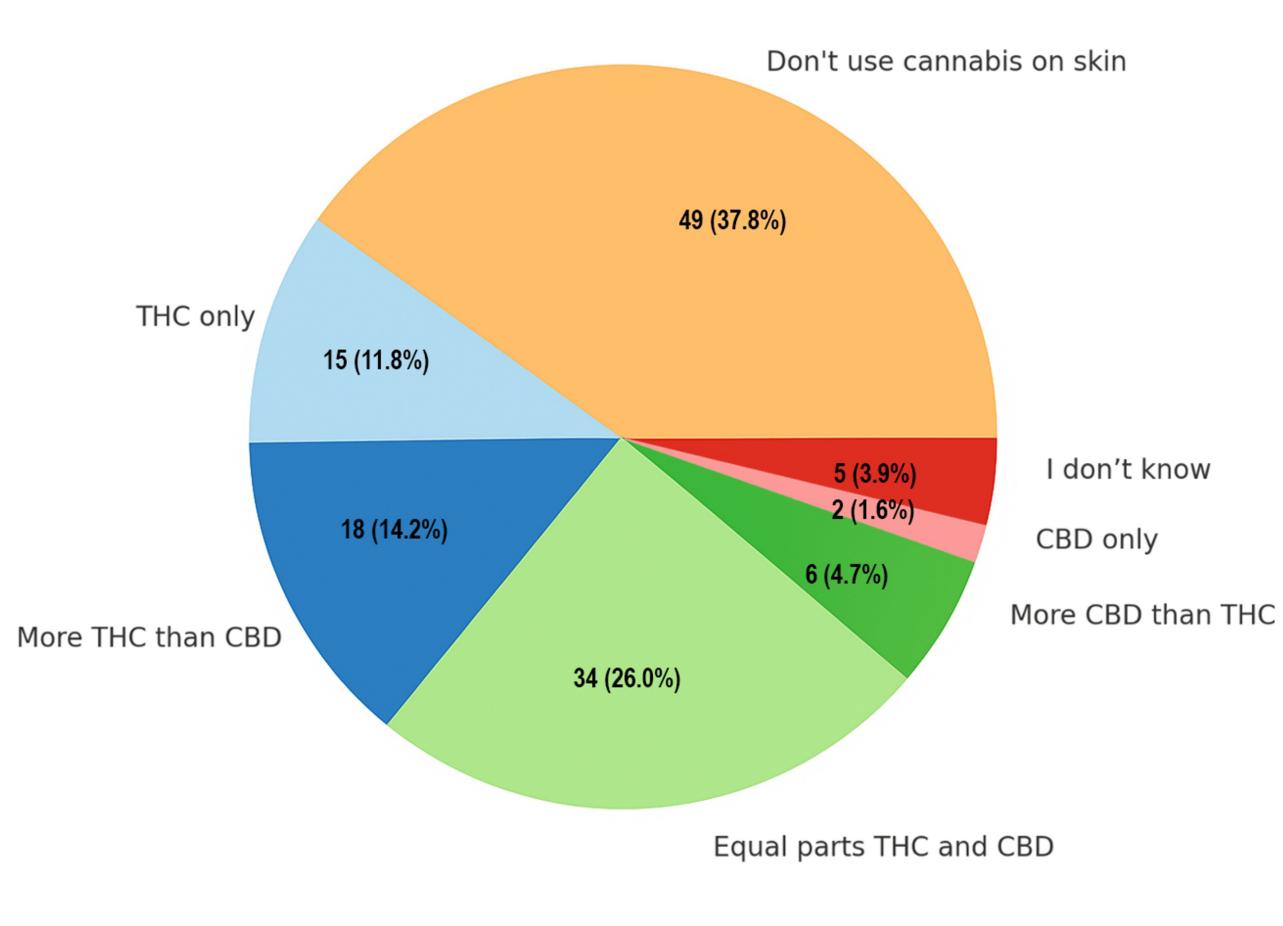
Composition of topical cannabis products used. Data are presented as the number and percentage of respondents (N, %). CBD: cannabidiol; THC: tetrahydrocannabinol.

A total of 49 respondents (37.8%) indicated that they do not normally use cannabis on their skin. Among those who do, 33 (25.9%) use products with equal parts THC and CBD, while 18 (14.1%) use products with more THC than CBD. A small portion of two respondents (1.5%) reported using CBD-only products (Figure 4).

**Figure 5:**
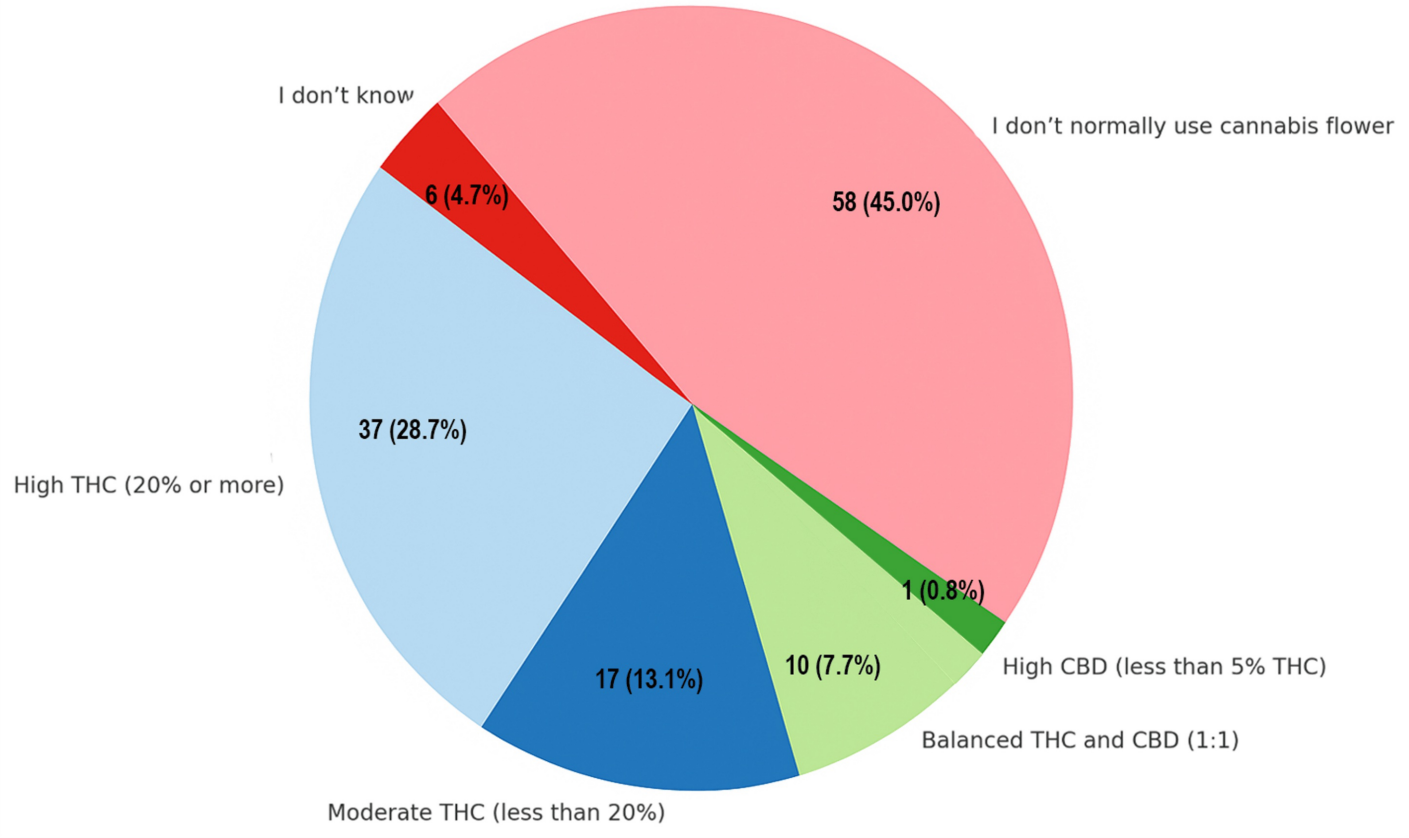
Preferred cannabis flower type. Data are represented as the number and percentage of respondents (N, %). CBD: cannabidiol; THC: tetrahydrocannabinol.

Among flower users, 37 (28.6%) used high-THC flower (≥20% THC), 17 (13.1%) used moderate-THC flower (<20%), and 10 (7.7%) used balanced 1:1 THC:CBD flower. The majority (58/129; 44.9%) did not use cannabis flower, and six (4.6%) were unsure of the type. No respondents used CBD-only flower, such as hemp (Figure 5).

**Figure 6:**
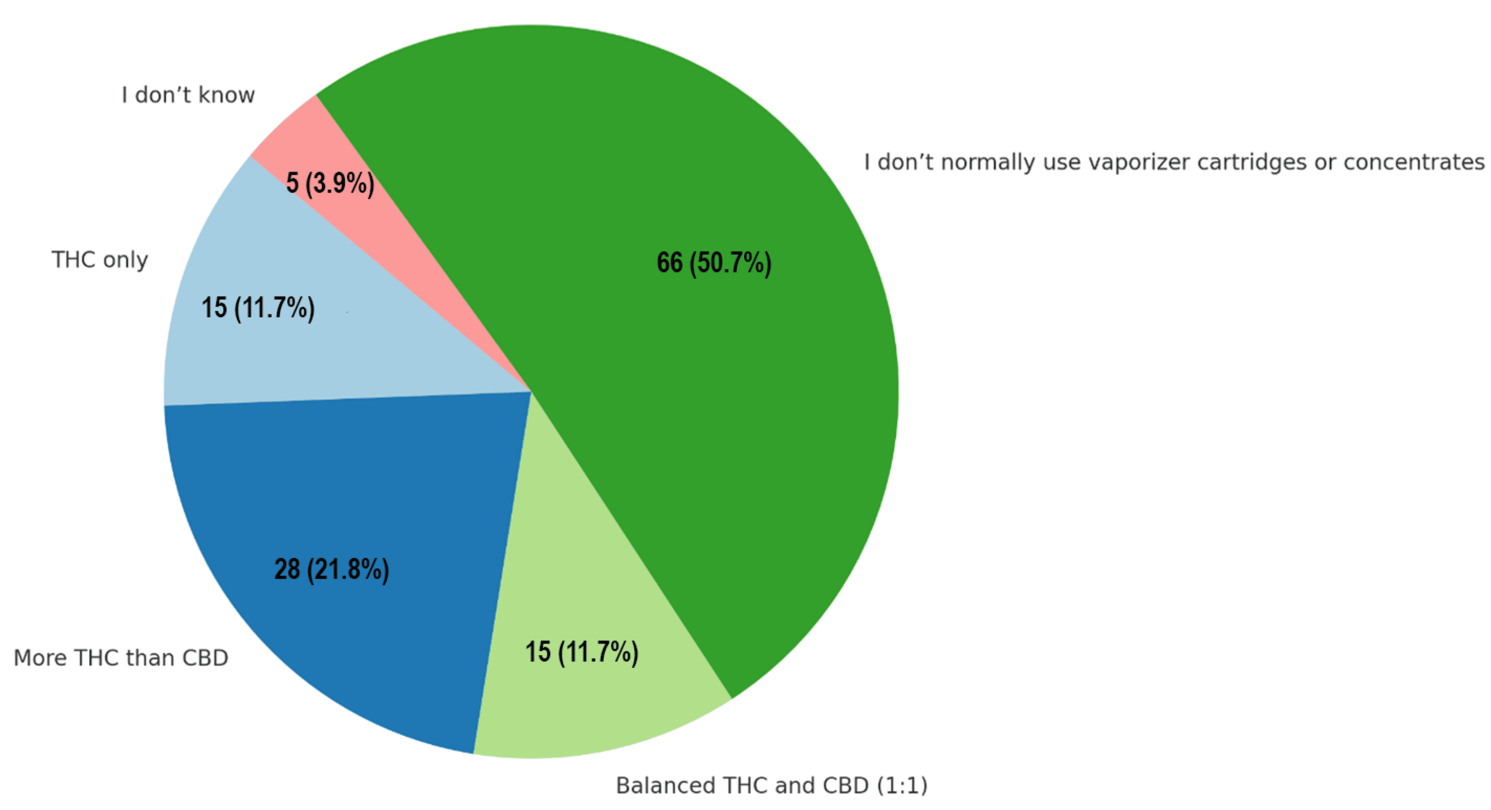
Composition of vaporized cannabis products. Data are shown as the number and percentage of respondents (N, %). CBD: cannabidiol; THC: tetrahydrocannabinol.

For respondents who vaporize cannabis oil or concentrates, 28 individuals (21.8%) most often used products containing more THC than CBD, while 15 (11.7%) used THC-only products, and another 15 (11.7%) preferred a balanced 1:1 THC to CBD formulation. A significant proportion, 66 respondents (50.7%), reported that they do not normally use vaporizer cartridges or concentrates. No participants reported using high-CBD or CBD-only products (Figure 6).

The majority of respondents expressed positive views on the effectiveness of cannabis in improving their main symptom, with 66 (51.1%) strongly agreeing and 55 (42.6%) agreeing with the statement. A small portion of seven (5.4%) were neutral, neither agreeing nor disagreeing, and only one respondent (0.7%) disagreed, suggesting that most respondents find cannabis beneficial for symptom relief.

**Figure 7:**
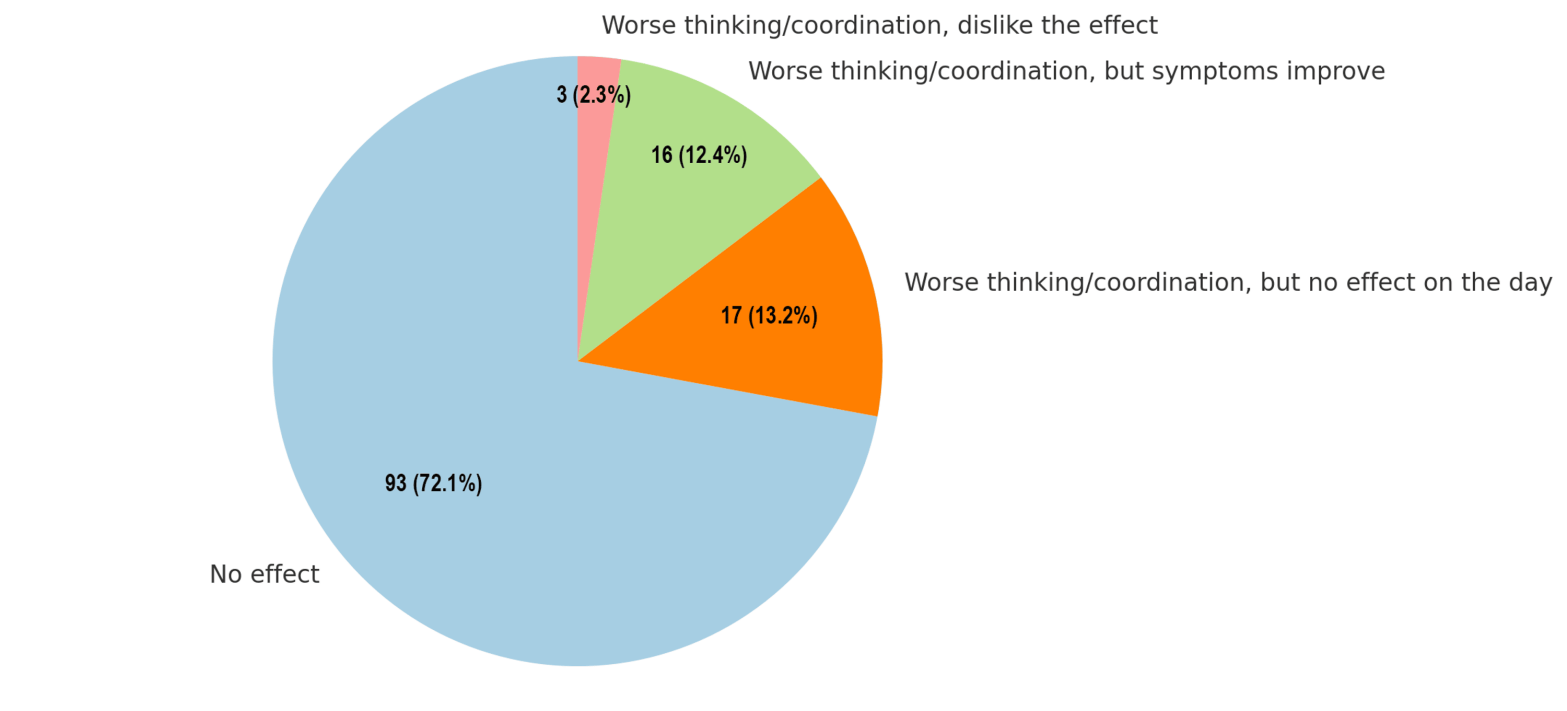
Effects of cannabis on thinking and coordination. Data are shown as the number and percentage of respondents (N, %).

Regarding the effects of cannabis on thinking and coordination, the majority (93, 72.1%) reported no effect on their cognitive or motor functions. A smaller proportion of 16 respondents (12.4%) experienced worse thinking and coordination but noted improvement in their symptoms. Conversely, 17 (13.2%) indicated worse thinking and coordination without any noticeable effect on their overall day. Only three (2.3%) expressed dissatisfaction, reporting worse thinking and coordination, and disliking the effect entirely. These findings highlight that while most individuals do not perceive any impact, a subset of users may experience varying degrees of cognitive or motor impairments, with some associating these effects with symptom relief. This distribution underscores the diverse responses to cannabis and the importance of individualized assessments in understanding its impact on cognitive and motor functions (Figure 7).

Regarding how cannabis use impacts mood and the feeling of being "high," out of 129 respondents, the majority reported that it changed their mood for the better, while a smaller percentage experienced no impact or undesirable effects (Table 1).

**Table 1:** Effects of cannabis on mood. Data are shown as N (%).

Response	Frequency	Percent
It changes my mood, but it makes my day even better	55	42.6%
It changes my mood, but it doesn’t impact my day	23	17.8%
It changes my mood, and I don’t like it (my day is worse)	2	1.5%
It changes my mood, and I don’t like it, but I put up with it because my symptoms are better	2	1.5%
Cannabis doesn’t affect my mood	47	36.4%

Sometimes people develop tolerance to THC and need to use more cannabis to help with their symptoms. Patients were asked to choose the answer that best matched how their cannabis use has changed over the last three months. These data show that 103 of the respondents (79.8%) reported no change in their cannabis use over the last three months, while only a small percentage needed to increase their dosage or frequency (Table 2).

**Table 2:** Changes in cannabis use due to tolerance over the past three months. Data are presented as N (%).

Response	Frequency	Percent
I needed more cannabis (larger amounts)	5	3.8%
I needed to use cannabis more often (more times per day)	4	3.1%
I needed more cannabis, AND I needed to use it more often	1	0.7%
My cannabis use has stayed the same	103	79.8%
My cannabis use has gone down	12	9.3%
N/A - I haven’t been using cannabis that long	4	3.1%

**Figure 8:**
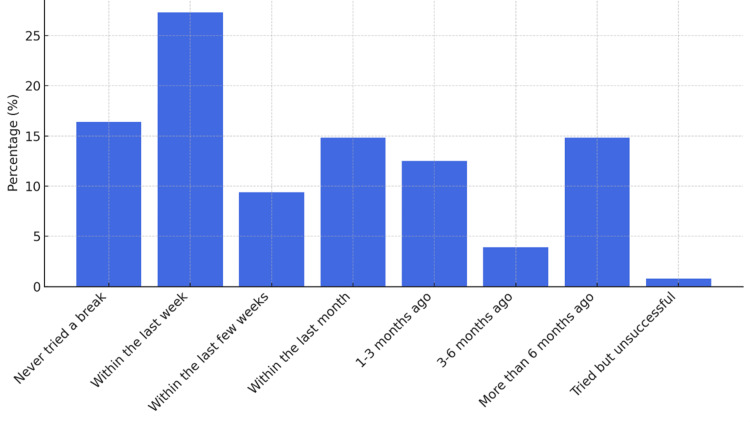
Time since last cannabis break (≥48 hours). Data are shown as the percentage of respondents (%).

**Figure 9:**
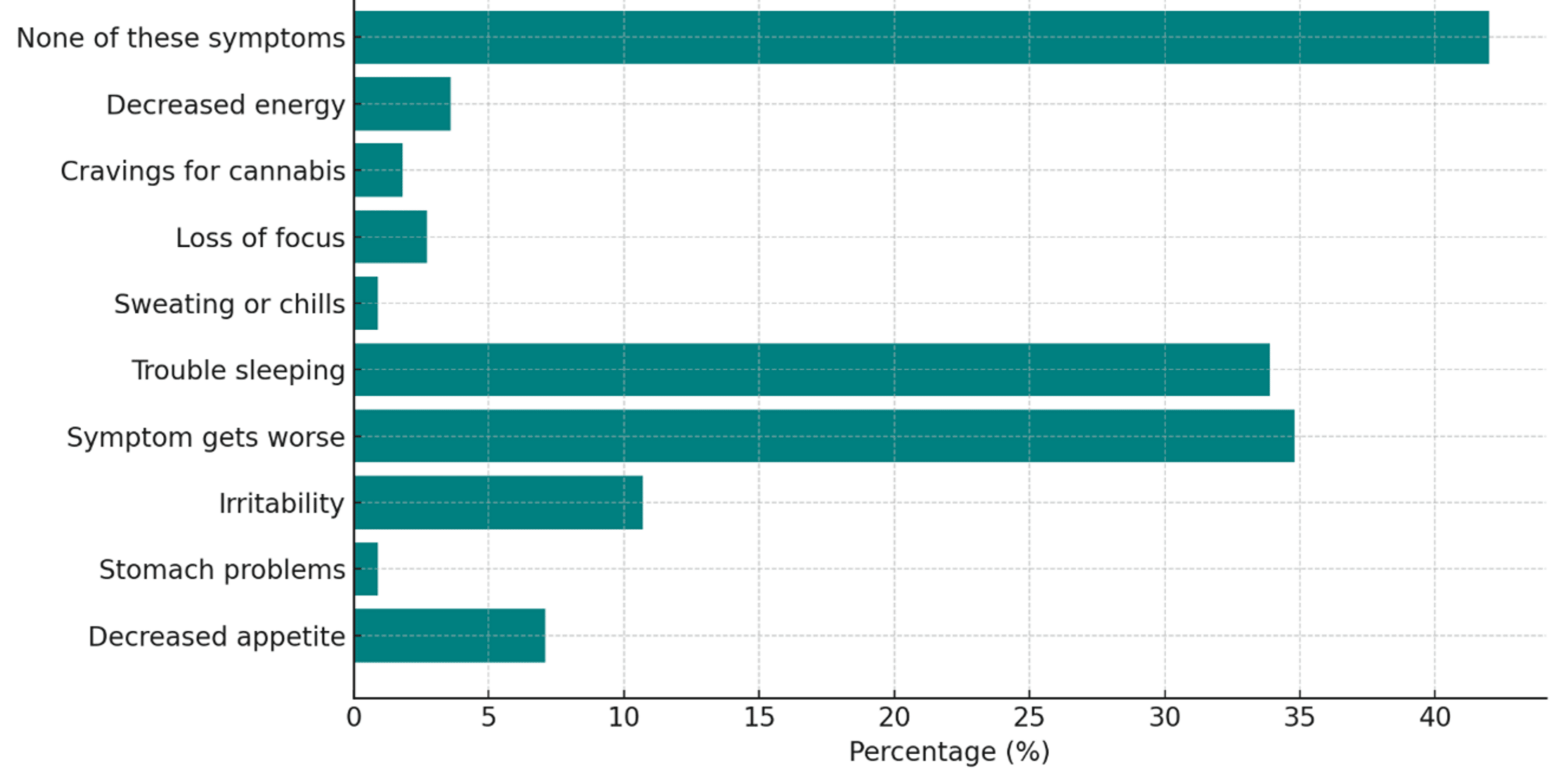
Reported symptoms when taking a break from cannabis. Data are presented as the percentage of respondents (%).

When asked how recently have they taken a break from cannabis for two or more days (48+ hours), the results showed that the largest group of 35 respondents (27.3%) had taken a break within the last week, while 19 (14.84%) had not taken a break in over six months (Figure 8). The most frequently reported symptom when taking a break from cannabis was a worsening of the initial condition for which cannabis was used, experienced by 45 participants (34.8%). Trouble sleeping was also a significant issue, affecting 44 respondents (33.9%). Despite these challenges, 54 participants (41.9%) indicated that they did not experience any of the listed symptoms (Figure 9). Other less commonly reported symptoms included decreased energy, loss of focus, cravings for cannabis, stomach problems, and sweating or chills.

When asked if they had ever felt the need to cut back on cannabis use while treating their main symptom, 111 of the respondents (86%) reported no such inclination, while a small proportion of seven (5.4%) indicated that they did feel the need to reduce their use. Similarly, when asked if others had ever suggested cutting back on their cannabis use, an overwhelming majority of 128 (99.2%) responded negatively, with only one respondent (0.8%) reporting that they had received such a suggestion. These findings suggest that most individuals do not perceive a need to limit their cannabis use for symptom management, nor do they commonly face external recommendations to do so.

## Discussion

Chronic musculoskeletal pain remains a leading cause of disability and diminished quality of life worldwide, often requiring long-term management strategies that balance efficacy, safety, and patient preferences. Amid increasing interest in alternative and adjunct therapies, MC has emerged as a commonly used option among patients seeking symptom relief with potentially fewer side effects than traditional pharmacologic treatments. However, data on patterns of use, preferred formulations, dosing, route of delivery, and cognitive effects remain limited. In this study, we utilized the IMCU questionnaire to assess real-world usage patterns, perceived efficacy, and cognitive impacts among 129 individuals using cannabis to manage chronic musculoskeletal pain. Our findings highlight that most participants favored daily administration, with topical applications, capsules, edibles, and tinctures being the most frequently employed methods. Although a majority reported symptom improvement, a substantial proportion lacked knowledge regarding the specific THC and CBD doses consumed, raising concerns about patient education and product dosing labels. Furthermore, while most respondents experienced no adverse cognitive effects, a subset reported impaired thinking and coordination, underscoring the need for individualized assessment and guidance in cannabis-based pain management.

The existing literature highlights short-term cognitive impairment as a notable concern associated with cannabis use. Additionally, users may experience behavioral and psychological changes, including but not limited to, heightened weakness, fatigue, dizziness, and a sense of intoxication [5-12]. More severe short-term adverse effects are often linked to higher concentrations of THC [6,7]. Studying these more severe side effects is crucial, given that cannabis use has shown associations with psychiatric and substance use disorders, particularly in individuals dealing with chronic pain [13,14]. This contrasts with the findings from the current study, as many patients denied effects on impairment and euphoria. This may be explained by the patients potentially using products with lower THC concentrations and topicals that are not intoxicating. However, this explanation cannot be confirmed with the current study due to patients’ inability to recall the dosages and/or concentrations of the products they were using and the lack of standardization of THC concentrations within and across studies.

When exploring the impact of MC on various aspects of patients’ daily activities, a substantial majority of patients perceived no negative effects. Specifically, over 70% reported no impact on their ability to engage in general activities, work or school tasks, household chores, running errands, driving, and caring for dependents. It is worth noting that some patients opted not to respond to these sections, potentially indicating a perception of unaffectedness. This suggests that the reported numbers might be even higher, underscoring the prevalent belief among patients that their daily lives remain largely unaffected by MC usage. The authors also believe that patients learn how to self-titrate to a THC dose they can function on and choose to use more intoxicating dosages when they are not driving or just relaxing before going to sleep.

Although a substantial number of patients claim no impact from MC on their ability to drive or operate machinery, this assurance falls short in a clinical context. Extensive research has consistently demonstrated the impairing effects of cannabis on driving performance, correlating its usage with heightened road traffic accident rates. Notably, across the UK, USA, Australia, New Zealand, and several European countries, cannabis, second only to alcohol, remains the most frequently detected substance among drivers involved in fatal accidents or stopped for impaired driving [9,10,15]. Ideally, achieving a unanimous 100% of patients reporting no impact from MC on driving ability would align with safety objectives.

When examining patient tolerance to MC, nearly half of the patients maintained consistent cannabis use over the past year without the need to increase the amount or dose to achieve the same relief for their main symptom. Regarding the side effects experienced during a break from MC, almost half of the patients reported no symptoms such as trouble sleeping, decreased appetite, loss of focus, irritability, sweating/chills, or cravings for cannabis. Around 34% of patients noted a worsening of their medical condition when abstaining from MC. It is important to note that some patients selected multiple side effects, potentially skewing the prevalence of reported symptoms upward compared to those who just selected no symptoms. One of the most significant findings highlights the overwhelmingly positive perceptions around cannabis use among patients. Nearly 99% of respondents reported that neither their friends, family members, nor healthcare providers had ever expressed concerns or suggested that their cannabis usage posed a problem.

There are several limitations to this study that could affect the reliability and interpretation of the presented findings. One limitation is self-reporting bias due to patients’ subjective perceptions and selective reporting, leading to varying interpretations and underreporting of certain side effects due to stigma or memory recall issues. Another limitation is the lack of control due to variability in dosage, strains used, and concurrent medications that can complicate the direct attribution of side effects to cannabis. Of note, the IMCU questionnaire utilized in this study asked about product dosing; however, nearly half the patients did not know, which could be attributed to poorly labeled products. Another limitation is potential sample biases, which might limit the generalizability of results, while the lack of objective measures and reliance solely on self-reported data could affect accuracy. Additionally, social stigma and legal concerns surrounding cannabis use may lead to underreporting of certain side effects and/or reporting accuracy. There may also be a response bias favoring either positive or negative reporting, influencing the study’s outcomes. Future studies may include long-term follow-up to report on delayed or cumulative side effects, enhancing the depth of their observations.

Despite these limitations, this study has key strengths that bolster its credibility and significance. By directly capturing patients’ experiences and perceptions, the study offers invaluable insights into how individuals navigate and perceive the side effects of MC in their daily lives. The comprehensive data collection covering a wide range of side effects provides a holistic understanding of the potential impacts of cannabis on patients’ well-being. Incorporating patient perspectives reflects a patient-centered approach, aligning with the aim of understanding and addressing patient needs and concerns, while also offering practical implications for clinical decision-making. Moreover, by contributing to the growing body of research on MC, this study fills critical gaps in understanding patient-reported outcomes, potentially influencing policies, regulations, and clinical practices in the realm of MC usage. Ultimately, these strengths collectively enrich our comprehension of patient experiences with MC and hold significant potential for informing future research, clinical guidelines, and healthcare policies in this domain.

## Conclusions

This study provides important insights into the real-world patterns, perceived efficacy, and cognitive effects of medical cannabis use among individuals with chronic musculoskeletal pain who employ cannabis regularly over extended periods. The results indicate that the users prefer non-inhaled formulations such as topicals, capsules, edibles, and tinctures. While the majority report meaningful symptom relief and minimal cognitive impairment, a significant proportion of users are unaware of the specific THC and CBD content in their products, pointing to a critical gap in patient education and product labeling. Additionally, although adverse cognitive effects were uncommon, their presence in a minority of respondents suggests the importance of individualized monitoring. These findings underscore the need for clinicians to engage in informed discussions with patients about cannabis use, promote accurate dosing awareness, and support further research to optimize safety and therapeutic outcomes in this population.

## Appendices

**Table 3:** Inventory of Medical Cannabis Use (IMCU) questionnaire. CBD: cannabidiol; THC: tetrahydrocannabinol.

Q#	Question	Response options							
	What is the main symptom you are treating with cannabis?								
	How long have you been using cannabis to manage your main symptom?	☐ 30 days or less ☐ 1-3 months ☐ 3-6 months ☐ 6-12 months ☐ More than a year ☐ About 2 years ☐ More than 2 years							
	On average, how often do you use cannabis?	☐ Once a month or less ☐ 2-3 times a month ☐ Once a week ☐ Twice a week ☐ Several times a week ☐ Once a day ☐ 2-3 times a day ☐ More than 3 times a day							
	How often do you use the following forms of cannabis?	Always		Often		Sometimes		Never	
	Flower or herb - Smoked								
	Flower or herb - Vaporized								
	Oil (cartridge or pen) - Vaporized								
	Concentrates (dabs, wax, shatter) - Vaporized								
	Oil or tincture - Under the tongue								
	Capsules, edibles, oil, or tincture - Swallowed								
	Topicals								
	If you take cannabis by mouth (edibles, tinctures, capsules, etc.), how much THC and/or CBD do you take at one time? Fill in the blank with the number of milligrams	THC: ________mg ☐ I don’t know how much THC I usually take. CBD: ________mg ☐ I don’t know how much CBD I usually take							
	If you apply cannabis onto your skin, how much THC and/or CBD is in the product you use most often?	☐ THC only ☐ More THC than CBD ☐ Equal parts THC and CBD ☐ More CBD than THC ☐ CBD only ☐ Other (please fill in): ____________________________ ☐ I don't normally use cannabis on my skin							
	If you use cannabis flower (raw plant matter), what kind of cannabis do you use most often?	☐ High THC (20% or more) ☐ Moderate THC (less than 20%) ☐ Balanced THC and CBD (for example, 1:1 ratio of THC:CBD) ☐ High CBD (less than 5% THC) ☐ CBD only (such as hemp) ☐ I don’t know ☐ I don’t normally use cannabis flower							
	If you vaporize cannabis oil or concentrates, what kind of product do you use most often?	☐ THC only ☐ More THC than CBD ☐ Balanced THC and CBD (for example, 1:1 ratio of THC:CBD) ☐ High CBD ☐ CBD only (such as hemp) ☐ I don’t know ☐ I don’t normally use vaporizer cartridges or concentrates							
	How much do you agree with this statement: “Cannabis improves my main symptom.”	☐ Strongly agree ☐ Agree ☐ Neither agree nor disagree ☐ Disagree ☐ Strongly disagree							
	Cannabis can affect your thinking and body coordination. Please choose a single answer that best matches how you usually feel after using cannabis:	☐ My thinking or coordination are worse, but it has no effect on my day ☐ My thinking or coordination are worse, and I don’t like it (my day is worse) ☐ My thinking or coordination are worse, but I put up with it because my symptoms are better ☐ My thinking or coordination aren’t affected by cannabis							
	Cannabis can change your mood or make you feel “high.” Please choose a single answer that best matches how you usually feel after using cannabis:	☐ It changes my mood, but it makes my day even better ☐ It changes my mood, but it doesn’t impact my day ☐ It changes my mood, and I don’t like it (my day is worse) ☐ It changes my mood, and I don’t like it, but I put up with it because my symptoms are better ☐ Cannabis doesn’t affect my mood							
	If cannabis affects your thinking, body coordination, or mood, which products cause this?	N/A	Thinking			Coordination		Mood	
	Flower or herb - Smoked								
	Flower or herb – Vaporized								
	Oil (cartridge or pen) - Vaporized								
	Concentrates (dabs, wax, shatter) - Vaporized								
	Oil or tincture – Under the tongue								
	Capsules, edibles, oil, or tincture - Swallowed								
	Topicals								
	Other (please fill in):								
	Sometimes people develop tolerance, and need to use more cannabis to help with their symptoms. Please choose the answer that best matches how your cannabis use has changed over the last three months	☐ I needed more cannabis (larger amounts) ☐ I needed to use cannabis more often (more times per day) ☐ I needed more cannabis, AND I needed to use it more often ☐ My cannabis use has stayed the same ☐ My cannabis use has gone down ☐ N/A - I haven’t been using cannabis that long							
	In the last three months, how often has your cannabis use (not your health condition) negatively affected your normal life activities such as:	Often			Sometimes		Rarely		Never
	General activity or hobbies								
	Work, school work, housework, or errands								
	Driving or operating machinery								
	Caring for children or dependents								
	Relationships with friends and loved ones								
	How recently have you taken a break from cannabis for two or more days (48+ hours)?	☐ I have never tried taking a break of 48+ hours ☐ Within the last week ☐ Within the last few weeks ☐ Within the last month ☐ 1-3 months ago ☐ 3-6 months ago ☐ More than 6 months ago ☐ I have tried taking a break, but I wasn’t successful							
	When taking a break from cannabis, do you notice any of the following symptoms? Check all that apply.	☐ Decreased appetite ☐ Irritability ☐ Trouble sleeping ☐ Loss of focus ☐ Cravings for cannabis ☐ Decreased energy ☐ Stomach problems ☐ My symptom (the reason I use cannabis) gets worse ☐ Sweating or chills ☐ None of these symptoms ☐ I have never taken a break from cannabis ☐ Other (please fill in):							
	While treating your main symptom, have you ever felt like you need to cut back on your cannabis use?	☐ Yes ☐ No ☐ I’m not sure or hard to say							
	While treating your main symptom, has anyone else suggested that you should cut back on your cannabis use? For example, a friend, family member, coworker, or healthcare provider?	☐ Yes ☐ No ☐ I’m not sure or hard to say							
